# Development of single-cell-level microfluidic technology for long-term growth visualization of living cultures of *Mycobacterium smegmatis*

**DOI:** 10.1038/s41378-021-00262-1

**Published:** 2021-05-20

**Authors:** Han Wang, Gloria M. Conover, Song-I Han, James C. Sacchettini, Arum Han

**Affiliations:** 1 Department of Biomedical Engineering, School of Medicine, Tsinghua University, 100084 Beijing, China; 2 Department of Biochemistry and Biophysics, Texas A&M University, College Station, TX 77843 USA; 3 Department of Electrical and Computer Engineering, Texas A&M University, College Station, TX 77843 USA; 4 Department of Biomedical Engineering, Texas A&M University, College Station, TX 77843 USA; 5 Present Address: Department of Medical Education, Health Sciences Center, Texas A&M University, Bryan, TX 77807 USA

**Keywords:** Engineering, Physics

## Abstract

Analysis of growth and death kinetics at single-cell resolution is a key step in understanding the complexity of the nonreplicating growth phenotype of the bacterial pathogen *Mycobacterium tuberculosis*. Here, we developed a single-cell-resolution microfluidic mycobacterial culture device that allows time-lapse microscopy-based long-term phenotypic visualization of the live replication dynamics of mycobacteria. This technology was successfully applied to monitor the real-time growth dynamics of the fast-growing model strain *Mycobacterium smegmatis* (*M. smegmatis*) while subjected to drug treatment regimens during continuous culture for 48 h inside the microfluidic device. A clear morphological change leading to significant swelling at the poles of the bacterial membrane was observed during drug treatment. In addition, a small subpopulation of cells surviving treatment by frontline antibiotics was observed to recover and achieve robust replicative growth once regular culture media was provided, suggesting the possibility of identifying and isolating nonreplicative mycobacteria. This device is a simple, easy-to-use, and low-cost solution for studying the single-cell phenotype and growth dynamics of mycobacteria, especially during drug treatment.

## Introduction

Tuberculosis (TB) sickens ~10 million people (5.6 million men, 3.2 million women and 1.2 million children) per year worldwide and killed an estimated 1.8 million people in 2020 (World Health Organization). TB is a major public health threat in part because the continuous emergence of multidrug-resistant bacteria gravely complicates the proper clinical management of the TB epidemic worldwide^1^. *Mycobacterium tuberculosis (Mtb)*, the causative agent of TB, possesses a multilayer cell wall that tightly regulates the transport of metabolites into the cell while ejecting antibiotics outside the cell wall. Notably, mycobacteria are capable of rapidly changing their growth rate depending on the available nutrients, making the bacteria highly effective at overcoming the immune response of its host cell^2^. Despite decades of study, the environmental and genetic triggers responsible for regulating the entry of *Mtb* into its nonreplicative state remain poorly understood. Hence, it is critical for investigators to elucidate the mechanisms used by this infectious pathogen to adapt its growth and render antibiotic treatments ineffective^3–5^.

Several studies have proposed that the nonreplicating growth state of mycobacteria may arise from a subpopulation of cells that successfully thrive for long periods of time in hypoxic conditions in host alveolar macrophages^6–8^. Transcriptomic or epigenetic changes are proposed to occur in the absence of genomic alterations; however, how this is accomplished remains particularly unclear for nonreplicating bacteria. While it might be feasible to kill drug-resistant bacteria with frontline TB antibiotics in some patients, it is much more difficult to manage the pathogenic potential of nonreplicating bacteria infecting lung macrophages in latent infections^3,9^. Despite the well-designed drug treatment strategies used in clinics, the biology of nonreplication remains elusive, making it very challenging for physicians and scientists to develop effective drug treatment regimens to treat active cases of drug-resistant TB^10–12^.

Changes in cell wall morphologies are often indicators of how mycobacteria will respond to certain drug regimens. Part of the challenge in such studies lies in the inability to monitor live mycobacteria for long periods of time under different culture conditions. For instance, the inability to examine bacterial growth under sequential rounds of antibiotic treatment while monitoring cell phenotype changes during extended periods of growth greatly impedes full pharmacological profiling of small-molecule test drugs^13^. Compared to other intracellular bacterial pathogens, mycobacteria have a distinct growth phenotype, forming sticky clusters with mycolic acid-rich membranes that constitute a formidable barrier to antibiotic treatment. Thus, changes in these morphologies are often indicators of how mycobacteria respond to certain drug treatments.

Using a microdevice that allows single-cell imaging, a previous study showed that the faster-growing daughter cells of mycobacteria are sensitive to antibiotics that target the cell wall synthesis machinery, while the slower-growing daughter cells are generally resistant to drug treatment^14^. Such single-cell analysis reveals that nonreplicative mycobacterial cells are likely critical in latent infections, even though they are thought to account for a minute amount of the entire bacterial population^15,16^. Hence, single-cell resolution live-cell microscopy imaging and analysis is critical in further understanding the behavior and biology of these tiny subpopulations of nonreplicative mycobacterial cells. Technologies that allow such long-term real-time visualization of *Mycobacterium* cultures to track the adaptation of nonreplicative mycobacteria to drug treatments can thus have a major impact in this field. Once a better understanding of the mechanisms used by mycobacteria to adapt to a toxic drug microenvironment is obtained, researchers will be able to develop next-generation biologic and pharmaceutical agents that can better manage patients with active multidrug-resistant TB.

To understand how nonreplicative growth arises in a mycobacterial population, it is necessary to systematically track the kinetics used by replicative and nonreplicative bacteria in response to stress. Typically, in the laboratory, this is achieved using 96-well microplate cultures where mycobacteria are exposed to different concentrations of bacteriostatic or bactericidal antibiotics in a checkerboard pattern. Previous time-lapse microscopy studies reported that single *Mycobacterium smegmatis* (*M. smegmatis*) cells regulate cell size by a constant growth mechanism that promotes growth size heterogeneity in response to environmental stress^17^. However, because mycobacteria are much thinner than mammalian cells or most bacterial cells and range from 3 to 5 µm in length, it is very difficult to bring mycobacteria into clear definite focus using an inverted epifluorescence microscope, while conducting a perfusion culture or applying drug treatment, making it extremely challenging to track single bacterium growth and replication over long periods of time.

Several microfluidic devices have been developed in the last decade, where single eukaryotic or prokaryotic cells are trapped within cell-trapping microstructures and imaged at high resolution, while allowing media with or without drugs to be perfused throughout a long culture period^18–20^. However, the vast majority of such microdevices are primarily designed for mammalian cell trapping and culture, and their designs and trapping mechanism are often not suitable for trapping significantly smaller and nonspherically shaped bacterial cells^21,22^. Several studies on single-cell bacteria show that their phenotypic heterogeneity in culture and drug response can be observed utilizing various physical cell-trapping microstructures^23–25^. However, as noted above, studying mycobacteria is significantly more challenging than culturing commonly used bacterial cells such as *E. coli* due to the asymmetric rod shape and the lipid-rich cell wall that leads to the formation of overlapping 3D cell clusters^26^. Other noncontact-based cell trapping methods, such as dielectrophoresis and acoustophoresis-based cell trapping, cannot easily trap or control cells that are smaller than a few micrometers in size, as such forces are proportional to the size of the target cells. In addition, single-cell-resolution microscopy requires cells to be within the same focal plane at high magnification, which is challenging for examining *Mycobacterium*.

In recent years, a few microfluidic devices that are capable of trapping and imaging *Mycobacterium* growth for several hours of observation have been developed. For example, Wakamoto et al. developed a sandwich-type microfluidic device to trap single *M. smegmatis* cells using a porous membrane and studied their cycles of death and division in the presence of isoniazid, a frontline antibiotic against TB^27^. Golchin et al. utilized a similar sandwiched clamp design with additional alginate gel to confine mycobacteria and study the single-cell heterogeneity of *M. smegmatis*^23^. However, there are several limitations to this design. First, it is cumbersome to build the microdevice because the pressure applied to clamp the sandwich structures needs to be fine-tuned every time the microdevice is assembled, to constrain the bacterial cells in a single focal plane without damaging the thin cover glass, resulting in poor control over the microdevice. Second, the permeability of the cellulose membrane used to trap the cells has a significant influence on the effect of drug treatment, as the membrane must be soaked in methanol overnight and thoroughly cleaned to become permeable. A study by Aldridge et al. utilized a single-layer microfluidic device to examine the single-cell growth of *M. smegmatis*^14^. In this device, two perpendicularly placed microfluidic channels with different heights were used, where the shallow culture channel array (0.8–0.9 μm in height) allowed cell growth in a monolayer within these channels and single-cell-resolution microscopy, while the deeper perfusion channels (65–70 μm in height) placed perpendicular to these shallow channels were used to provide culture media to those shallow cell-culture channels. Using this apparatus, the authors reported unipolar growth and asymmetric division of *M. smegmatis* within four generations, where varying levels of cellular responses to antibiotics were found in asymmetrically dividing daughter cells. Despite its success, this microdevice design restricted the direction of growth of single bacteria in one dimension since the width (7 μm) of the channel allowed cell growth in only one dimension, exerting an additional mechanical stress that may affect the physiology and growth of the bacteria. The duration of the culture was also limited, since as cells divided, they escaped out of the shallow channel region, at which point further imaging was not possible. Making the shallow channel length longer is one possible way to increase the culture duration but could limit the perfusion of culture media into the shallow cell culture channel.

In this study, we advanced microfluidic technology to enable high-resolution visualization of the continuous growth and division dynamics of *Mycobacterium* and used this device to record morphological alterations in real time using live time-lapse microscopy for up to 48 h. This device design utilizes a dual-channel-height planar microfluidic structure that allows single *M. smegmatis* cells to be trapped in shallow microfluidic channel regions, and perfusion culture medium to pass through deeper microchannels connecting these shallow channels. Our design overcomes many challenges associated with the previously developed microfluidic systems for mycobacterial culture and imaging. Because *Mtb* is a biosafety level 3 respiratory pathogen with a very slow growth rate (several weeks of in vitro culture is typically required), in this study, we employed the fast-growing noninfectious model strain *M. smegmatis*. Using our device, we report, as a proof-of-concept, that single-cell growth in a single focal plane can be achieved and the growth/morphology monitored over a 48 h period during perfusion culture and drug treatment. This microfluidic device can be utilized to answer questions on how *Mtb* undergoes changes in its rod shape in response to antibiotic treatment and has the potential to facilitate future studies on isolating nonreplicative bacteria and conducting genomics/transcriptomics studies on such bacteria.

## Results

To perform long-term culturing of *M. smegmatis* at single-cell resolution, there are three key requirements for the microfluidic device. First, the device has to trap rod-shaped *M. smegmatis* cells that are typically <1 μm in height and 2–4 μm in length for consistent focused high-resolution (typically ×100 magnification) imaging. Second, to accurately document changes in cell phenotype throughout a given culture period, cell growth needs to be limited to a two-dimensional (2D) plane to maintain cells within the same focal plane and allow high-resolution microscopy across multiple generations. Third, the location of the cells needs to be relatively fixed over time rather than moving around freely in culture media to allow effective time-lapse tracking of individual cells.

**Fig. 1:**
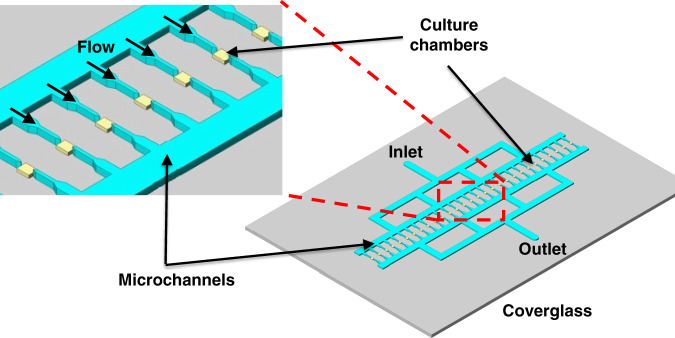
Microfluidic device design and specifications. The microfluidic device is designed for assessing *M. smegmatis* cell trapping, growth, and drug treatment for continuous live kinetic growth studies. This device is composed of arrays of *M. smegmatis* trapping/culture chambers that are only 0.9 μm in height (yellow box) and connected through arrays of deeper (14 μm in height) perfusion channels (cyan color). A polydimethyl siloxane (PDMS) microchannel layer is bonded onto a thin cover glass to form the microfluidic device.

Our microfluidic device consists of an inlet, an outlet, an array of 29 perfusion microchannels, and an array of 29 shallow culture chambers placed after the perfusion microchannel array. The perfusion microchannels (14 μm in height) were designed to be deeper than the culture chambers (0.9 μm in height) to continuously or periodically allow media and drugs to flow to the cells. These microchannels are also used to load cells into the microfluidic device and direct cells toward the shallow culture chambers (Fig. 1). Loaded *M. smegmatis* cells can be trapped in shallow culture chambers (460 μm × 300 μm in size) for all cells to be in a single focal plane, enabling high-resolution imaging. Due to the 2D nature of the culture chambers, even though bacteria grow toward the empty space in the shallow culture chamber, their growth is restricted in the Z-direction by the limited channel height, which allows individual growth tracking and cell morphology monitoring through the culture. Notably, the height of the outlet channel following the cell trapping and culture region was set to 14 μm, the same height as the main perfusion channel, to minimize the overall flow resistance in the microchannels by minimizing the length of the shallow microfluidic channel region.

Because *Mtb* cells grow extremely slowly in a laboratory setting (doubling time of ~8 weeks) and are a biosafety level 3 respiratory pathogen with strict containment requirements, the fast-growing noninfectious.

*M. smegmatis* with a short doubling time in nutrient-rich conditions of 2–6 h was selected for this proof-of-concept study^28^. To study growth dynamics in long-term cultures, a mycobacterial expression plasmid-replicating codon-optimized mEmerald (co-Emerald) green was used to transform *M. smegmatis*. After cell trapping was achieved and verified by observation under the microscope, the regular culture media (7H9-ADC) was switched to media containing either a small-molecule test drug (methyl (4-{[(2,4-dimethylphenoxy) acetyl] amino} phenoxy) acetate) or the frontline anti-TB drug rifampicin. To track and identify nonreplicative bacteria and examine their capability to resume active growth after antibiotic treatment, their dynamic phenotypic changes were monitored under defined media schemes.

The bacterial strain was grown in a shaker incubator for ~16 h at 37 °C in 7H9-ADC media. A single-cell suspension was obtained through filtration and centrifugation of bulk culture and then loaded into the microdevice. Continuous perfusion culture was performed at 5 μl/h with time-lapse microscopy imaging while the microfluidic device was within an incubation enclosure of a microscope. Dead bacterial cells were stained by propidium iodide (PI) added to the media prior to loading the cells into the microdevice.

**Fig. 2:**
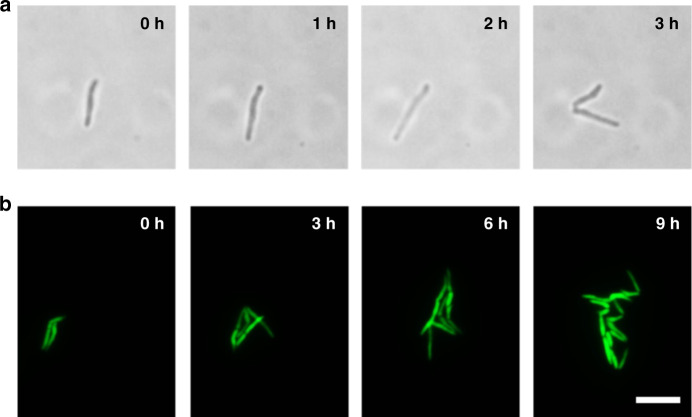
Long-term co-Emerald *M. smegmatis* live cell division and growth monitoring using the developed microfluidic device. **a** A single *M. smegmatis* cell was trapped and cultured inside the microdevice, showing asymmetric division within 3 h. **b** Fluorescence images show two *M. smegmatis* cells trapped initially but still demonstrate that the cells were dividing and growing only in 2D space without major cell overlap while maintaining sharp definite focus. These findings demonstrate the single-cell resolution culture and monitoring capability of the developed microfluidic device. Scale bar = 5 µm.

Co-Emerald-expressing *M. smegmatis* cells were loaded into the microfluidic device, and their growth was tracked over a 9 h period in 7H9-ADC media without additional drugs or antibiotics (Fig. 1). Bright-field images of a single *M. smegmatis* cell trapped in the chamber of the microdevice show that the cell was capable of division during a 3 h period (Fig. 2a). A single division cycle occurring within 3 h was observed, where the cells demonstrated asymmetric division, similar to that reported by previous studies^14^. As noted above, *M. smegmatis* is a fast-growing mycobacterial strain compared to the slow-growing pathogenic *Mtb* strain and, in favorable media conditions, typically reaches the stationary phase within 24 h. The culture in the microdevice showed that *M. smegmatis* cells grew and spread only in 2D space in the shallow microchambers, with almost no overlapping cells clumping on top of one another (Fig. 2b). Throughout the 9 h culture period, most cells remained in sharp focus despite the high magnification used (×100 magnification), and cells, for the most part, stayed in the same position during continuous perfusion of the culture media. This initial result shows that the device enables monitoring if morphological changes in cells during culture and that it can be used to quantify the lengths of individual cells throughout the culture period.

**Fig. 3:**
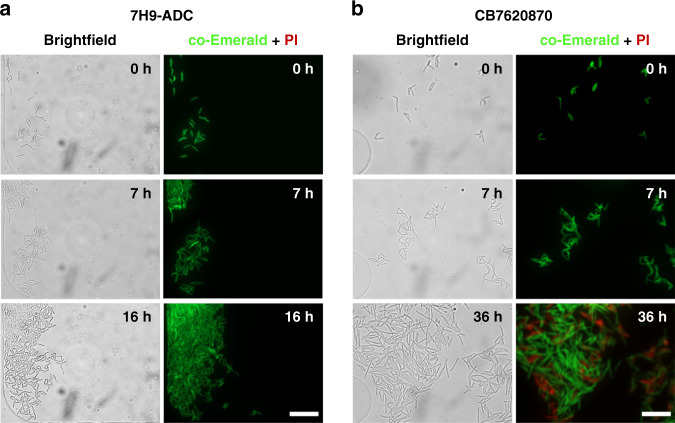
Simultaneous tracking of bacterial growth kinetics in two parallel channels with two different culture conditions. The growth kinetics of co-Emerald *M. smegmatis* cells treated with the small-molecule test drug CB7620870 compared to cells growing in normal media 7H9-ADC were compared. **a** *M. smegmatis* cells cultured in 7H9-ADC exhibited robust exponential growth with minimal cell death, as evidenced by the increase in live *M. smegmatis* cells expressing co-Emerald and the absence of PI-stained *M. smegmatis* cells during the 16 h culture period. **b** *M. smegmatis* cells treated with the small-molecule test drug CB7620870 for 29 h at 200 μM after being cultured initially in 7H9-ADC for 7 h showed significantly slower growth, with some cell death indicated by PI staining. Scale bar = 20 µm.

To visualize how the growth behavior of *M. smegmatis* changes in the presence of a small-molecule test drug, the growth of *M. smegmatis* cells in either 7H9-ADC media alone or in media containing the small-molecule test drug CB7620870 was compared in cultures grown in parallel microchannels of the same microfluidic device. CB7620870 is a small-molecule test drug with a molecular weight of 343 g/mol and a pKa of 12.7 that inhibits the proper targeting and function of the bacterial membrane protein Wag31 (Rv2145c)^29^. Bright-field and fluorescence images of *M. smegmatis* cells cultured in 7H9-ADC media show exponential growth inside the device for several hours and that the cells readily formed a large cell cluster at 16 h, although the cells mostly remained as a focused monolayer (Fig. 3a), demonstrating that the microdevice indeed enables the cells to grow in a 2D monolayer as intended. In contrast, when exponentially growing *M. smegmatis* cells were treated with CB7620870 (200 µM) at the 7 h time point, the growth rate was substantially reduced, where cell clusters could be observed only at ~36 h (Fig. 3b, 36 h, vs. Fig. 3a, 16 h). *M. smegmatis* cells stained with PI dye (red) showed some dead cells after treatment with the small-molecule test drug, but no dead cells were observed during normal culture in 7H9-ADC media.

**Fig. 4:**
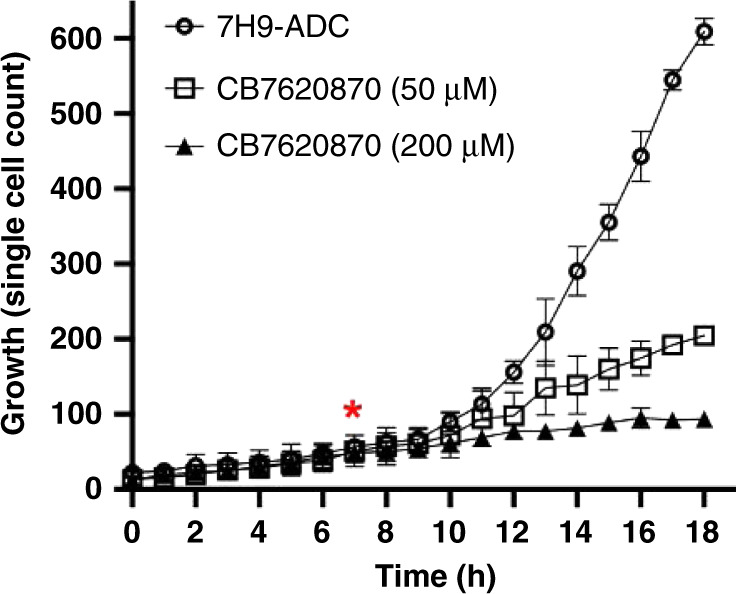
Comparison of the growth dynamics of *M. smegmatis* in the microfluidic device. Single-cell growth inside the multichannel microfluidic device was observed for 18 h, and images were obtained hourly. *M. smegmatis* cells were loaded into 3 parallel microchannels and allowed to grow for 7 h undisturbed. At this time point (*t* = 7 h, denoted by the red asterisk in the graph), no drug was added to channel 1, but CB7620870 was added to channel 2 at a 50 μM concentration and to channel 3 at a 200 μM concentration. There was an ~2–3 h delay in the observed changes in the growth rate (comparing the cell numbers of drug-treated cells [squares and triangles] to those of untreated cells [circles]).

To better understand the growth characteristics of *M. smegmatis* inside the microfluidic device compared to those in regular flask culture, the growth dynamics were compared (Fig. S1). Bacterial growth in 5 ml cultures was monitored inside small shaker flasks by taking optical density readings at 600 nm for 7 days. The results obtained with this approach indicate that the culture grown in 7H9-ADC media reached the saturation phase ~22 h after inoculation. When monitoring *M. smegmatis* growth inside the microfluidic device through time-lapse imaging, the bacterial cells began dense logarithmic growth after ~9 h (comparison of the growth kinetics in Fig. S1 vs. Fig. 4). In parallel, for drug treatment studies, the bacterial cells were allowed to grow undisturbed for 7 h in the microfluidic device, at which point the test drug CB7620870 was added at a concentration of 50 μM or 200 μM (Fig. 4). Our analysis shows that it takes ~3 h until cellular growth is notably suppressed in response to drug treatment. In conclusion, these data show that despite being confined in 2D space inside the microfluidic device, the growth dynamics (i.e., growth rate) of *M. smegmatis* are similar to those observed in bulk culture, allowing the benefit of single-cell-resolution monitoring of cell growth and phenotypic changes under normal and drug treatment conditions.

**Fig. 5:**
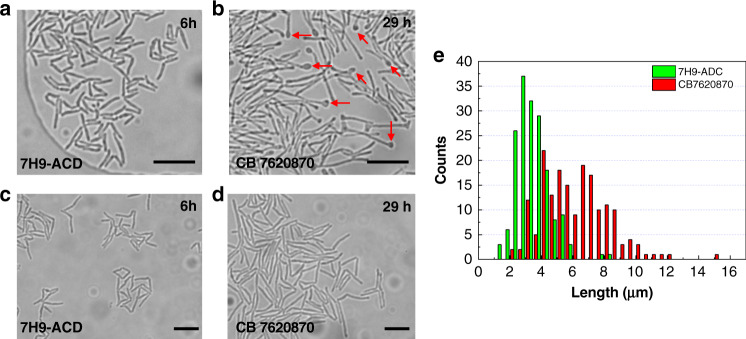
Occurrence of a knob-like membrane protrusion at the bacterial poles and increase in the cell length upon drug treatment in the microfluidic device. Bright-field images of live *M. smegmatis* cells (**a**) cultured in 7H9-ADC for 16 h compared to (**b**) those cultured in CB7620870 for 29 h. In the drug treatment group, cells were allowed to grow for 7 h in 7H9-ADC media first and then treated with 200 µM drug for 29 h. Drug-treated cells showed clear knob-shaped swelling at the poles (red arrows). Microscopy images showing the *M. smegmatis* cell length change when (**c**) grown in 7H9-ADC for 6 h and when (**d**) exposed to CB7620870 for 29 h, displaying an elongated shape after treatment. **e** Histogram of *M. smegmatis* cell length changes after exposure to the drug CB7620870 (9.2 ± 4.9 μm) and when cultured in normal media 7H9-ADC (4.3 ± 1.9 μm). Scale bar = 5 µm.

In addition to the slower growth rate and increase in the number of dead cells when *M. smegmatis* cells were treated with CB7620870, the results show that the cells exhibited rather dramatic morphological changes at the poles of their cell wall. After drug treatment, *M. smegmatis* revealed a gross malformation, displaying a knob-like structure, and showed an increase in the number of elongated thin rod-shaped cells (Fig. 5a shows cells cultured in 7H9-ADC media alone, and Fig. 5b shows cells treated with CB7620870 for 29 h at 200 μM after 7 h of culture in 7H9-ADC media). It is clear that CB7620870-treated *M. smegmatis* cells show distinct phenotypic changes where the poles of cells are notably deformed, showing large knob-like poles in the elongated cells (Fig. 5b arrows). These results suggest that this drug may have interfered with the formation of a membrane protein complex structure that governs bacterial elongation and division. Such an observation is not possible without the single-cell resolution imaging throughout the drug treatment period enabled by the microfluidic device.

Another major observation in this study is the significant increase in the bacterial rod length upon exposure to the drug CB7620870. Image analysis conclusively reveals that the average bacterial length becomes significantly longer when exposed to the small-molecule test drug CB7620870 (Fig. 5c vs. 5d). The bacterial length distribution was quantified and graphed as a histogram (Fig. 5e) using a custom MATLAB^TM^ image-processing algorithm. Our results show the emergence of abnormally long cells, as the average bacterial length nearly doubled from 4.3 ± 1.9 μm (*N* = *55*) to 9.2 ± 4.9 μm (*N* = *198*) after CB7620870 treatment (*p* < 0.05, *p* = 1.39E-23). Interestingly, the cell length distribution was significantly broader when the cells were treated with this drug, showing significantly high heterogeneity. Taken together, these results show that *M. smegmatis* fails to divide normally in the presence of CB7620870, suggesting that long-term exposure to this drug perturbs bacterial cell division.

**Fig. 6:**
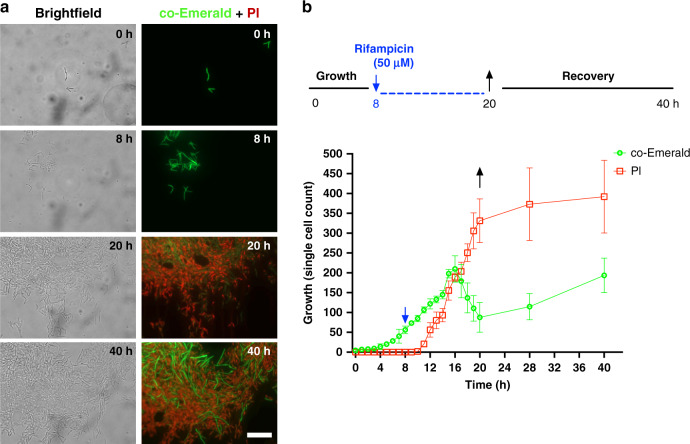
Real-time visualization of fast-emerging replicating *M. smegmatis* cells after removal of the antibiotic rifampicin from the culture, suggesting the existence of a persistent *M. smegmatis* phenotype. **a** Bright-field and fluorescence micrographs of live *M. smegmatis* expressing the co-Emerald plasmid in a microfluidic device show normal exponential growth (0–8 h), followed by massive cell death after antibiotic exposure (after 8 h). The surviving bacterial population recovered, and growth re-emerged within 20 h (i.e., increased appearance of *M. smegmatis* expressing the co-Emerald plasmid at the 40 h time point). Scale bar = 5 µm. **b** A graph showing the relative number of live (co-Emerald, green) and dead (PI, red) *M. smegmatis* cells over time. The blue arrow shows the time point at which rifampicin was added, while the black arrow indicates the time point at which rifampicin was replaced with 7H9-ADC media. The number of cells counted for the graph reflects cells that were visible within the field of view (80 µm × 110 µm).

Next, we analyzed how *M. smegmatis* cells behave when treated with the frontline antibiotic rifampicin, a drug commonly used in the clinic to treat patients with chronic and drug-resistant TB, using the developed microfluidic device. As before, *M. smegmatis* cells were loaded into the microfluidic device and allowed to adapt to grow in the device in 7H9-ADC media alone for 8 h. In the control group cultured only in 7H9-ADC media, *M. smegmatis* cells grew exponentially, similar to the result shown in Fig. 4. In the drug treatment group, rifampicin (50 µM) was loaded into the microfluidic device at the 8 h time point, and the cells were continuously treated for 12 h. After the drug treatment, most cells were observed to be dead, as quantified by counting cells that had lost their co-Emerald plasmid expression and stained with PI (20 h time point, Fig. 6a). However, a small number of surviving cells were observed even after rifampicin treatment. Interestingly, after switching back to 7H9-ADC media for another 20 h of culture, it was observed that the surviving *M. smegmatis* expressing co-Emerald started to grow again, eventually achieving a large population that saturated the entire culture chamber region of interest. Thus, we concluded that a persistent *M. smegmatis* phenotype was observed. Note that due to the slow perfusion culture flow rate (5 μl/h) and relatively long tubing length from the syringe pumps placed outside of the microscope incubator, it takes 1.5 h for the drug solution to enter the microdevice from the syringe pumps. This is reflected in the delay seen between the time indicated as the drug treatment start time and the actual time that the cells were exposed to rifampicin (Fig. 6b). A faster flow rate would increase the shear stress and affect the perfusion culture as well as increase the clump formation of mycobacteria and thus was not applied.

## Discussion

Single-cell-resolution phenotyping of live mycobacterial growth kinetics is a crucial first step toward the full mechanistic understanding of how a small subpopulation of mycobacteria adapts and adjusts their growth behavior in real-time in response to antibiotic exposure. In this study, two major technological advancements in microfluidic device design were achieved for single-cell-resolution microscopy of live mycobacterial cells. First, we successfully immobilized mycobacterial cells for long-term single-cell resolution visualization under perfusion culture conditions for over 48 h. Second, we were able to maintain mycobacterial cell growth in a single focal plane for the high-resolution imaging that is needed to observe detailed phenotypic changes of mycobacterial cells. In general, high-resolution imaging of single bacterial cells that are in suspension and not adhered to a surface cannot be achieved unless there is a physical structure that holds down the cells in a single focal plane. For mycobacterial cultures specifically, an added challenge is that these cells typically grow into 3D microcolonies. These challenges are also evident from the limited reports of single-cell-level visualization of *Mycobacterium* over long periods of time despite the importance of better understanding of the persistence of *Mycobacterium*.

Our novel microfluidic culture device is composed of arrays of 29 mycobacterial cell trapping and culture chambers, enabling parallel and high-throughput phenotyping of mycobacterial cells. Our data shows that the developed microfluidic *Mycobacterium* culture microdevice enables consistent long-term visualization of *M. smegmatis* cells at single-cell resolution, enabling unprecedentedly detailed analysis of the bacterial growth dynamics during continuous perfusion culture (Fig. 2). Furthermore, our work shows that the microfluidic mycobacterial cell culture design effectively restricts *Mycobacterial* growth in a 2D space, significantly decreasing the 3D clumping tendency of mycobacterial cells that is often observed in shaker flask cultures. Thus, the microfluidic device makes it possible to keep the majority of the mycobacterial culture within a single focal plane, enabling high-resolution imaging over a continuous 48 h period (Figs. 3–6). This work significantly improves upon microfluidic technology for mycobacteria phenotypic observation under heterogeneous culture environments, enabling a broader understanding of how mycobacteria change their growth rate and adapt to specific drugs and how the cells modify their membrane structure upon drug exposure. In addition, these studies will permit future investigations to assess when and how persistent subpopulations arise when certain antibiotic combinations are used in a clinical laboratory. This knowledge is particularly critical for patients with drug-resistant TB bacterial strains.

From the perspective of microfluidic devices for mycobacterial culture and characterization, the presented microdevice design allows long-term single-cell growth assessments of four different culture conditions using a parallel channel design. Compared to the device reported by Wakamoto et al.^27^, instead of using a top perfusion channel layer and bottom cover glass layer separated by a porous cellulose membrane where cells are dispensed and then tightly clamped together by a metal fixture to restrict mycobacterial growth to one dimension, the presented microfluidic device affords precise control over the channel dimensions and minimum device-to-device variation. This is because the height of the mycobacterial culture chamber (0.9 µm), a crucial parameter that enables cell loading while restricting cell growth to only 2D, is set by the design of the microfluidic device itself, whereas in the previous design, it must be set by adjusting the mechanical clamping force that controls the chamber height. The *M. smegmatis* cells cultured in our microfluidic device showed a division cycle of 3 h, much closer to the typical doubling time of 3–4 h seen in bulk culture than the 6 h doubling time observed in the previous design, which suggests a potential negative influence on cell growth from the clamping device itself.

Compared to another previous work by Aldridge et al., the microfluidic device design presented here allows long-term culture and observation^14^. In this previous work, a narrow channel restricted the growth of *M. smegmatis* cells and enabled monitoring of the bacterial cells with single-cell resolution. However, the culture duration achieved in that work was only ~4 generations of cell division. Beyond that, the divided cells would simply grow out of the narrow channel and escape into the flow; thus, the observation was limited to ~12 h. This is because of the relatively short length of the culture channel, which can only hold a limited number of cells. A longer channel that can hold more cells, thus enabling a longer culture duration, limits media diffusion to the center of the microfluidic channel due to the narrowness of the microchannel that is needed to hold single cells. Overall, the 4-generation culture period limitation of the previous design may not be sufficient to examine cell-to-cell heterogeneity in cellular morphology and drug responses, as seen in the data presented here, where it took 29 h to see morphological changes of high interest, such as knob-like feature formation upon drug exposure. Our microfluidic device overcomes most of these challenges. Notably, our device is capable of robust 2-day single-cell *M. smegmatis* culture and live cell imaging analysis under varied culture conditions, including drug treatment and media exchange.

From the biological perspective related to *M. smegmatis* culturing using our microfluidic device, we observed that dramatic morphological changes (both in shape and length) occurred when these mycobacteria were treated with the small-molecule test drug CB7620870. Specifically, we observed a clear morphological change leading to significant swelling at the poles of the bacterial membrane, with a knob-like abnormality appearing after 29 h of exposure to a small-molecule test drug (Fig. 5). Significant changes in cell length and the heterogeneity in the length distribution were observed when cells were treated with this small-molecule test drug in long-term culture. Such morphology changes and heterogeneity can only be monitored through single-cell-resolution imaging, as provided by the presented microfluidic device. In addition, when mycobacteria were treated with the clinical frontline antibiotic rifampicin, we observed that a small subpopulation of cells survived the drug treatment. This small subpopulation recovered and achieved robust replicative growth once regular 7H9-ADC media was provided. These data strongly suggest that the emergence of nonreplicative or persistent *M. smegmatis* is readily reproducible in the laboratory. Ease in switching media during continuous single-cell-resolution imaging of mycobacteria is also a unique capability enabled by the developed microfluidic system. This finding also suggests the possibility that isolation of this subpopulation of bacteria may be feasible, enabling future studies to target the underlying biology of persistent bacteria in the context of different antibiotic regimens.

In conclusion, our study demonstrates that the newly developed microfluidic device is capable of successful long-term growth and phenotype monitoring of rod-shaped mycobacteria. We expect that this microfluidic device technology will substantially change how clinicians and scientists can identify the correct sequence and combination of antibiotics to tailor the treatment of TB patients to significantly reduce treatment relapses or death.

## Materials and methods

### Microdevice fabrication

The single-cell-resolution *M. smegmatis* culture microdevice was fabricated in poly(dimethylsiloxane) (PDMS, Sylgard^®^ 184, Dow Corning, Midland, MI) using a soft lithography technique. The two-layer master mold fabrication steps are similar to a microfabrication procedure we used previously for a neuron cell culture microdevice^30^. In brief, the master mold was patterned using multilayer photolithography to obtain microstructures with two different heights. First, the shallow cell culture chamber part was fabricated by spin coating a SU-8^TM^ 2005 photoresist layer to a thickness of 0.9 μm, followed by photolithography. Then, the deeper perfusion channel layer was fabricated by spin coating a SU-8^TM^ 2015 photoresist layer to a thickness of 14 μm, followed by aligning the perfusion channel layer pattern with the first culture chamber layer pattern. This master mold pattern was then transferred to a PDMS layer, which was then bonded to a 0.17 mm thick cover glass after oxygen plasma treatment (Expanded Plasma Cleaner, Harrick Plasma, Ithaca, NY). The fabricated microdevice was sterilized with UV light overnight and rinsed with PBS before loading cells into the microdevice and starting the culture and microscopy processes.

### *M. smegmatis* preparation and culture in the microdevice

A mycobacterial expression plasmid-replicating tool co-Emerald with a constitutive P38 promoter and a synthetic ribosomal binding site, which was C-terminally tagged with FLAG tag, was used to transform *M. smegmatis* mc2 155 ATCC 700084 (a kind gift from Prof. C. Sassetti, University of Massachusetts). A constitutively co-Emerald-expressing strain of *M. smegmatis* was grown in a shaker incubator from a glycerol stock source for ~16 h at 37 °C in Middlebrook culture media 7H9-ADC (Sigma-Aldrich, St. Louis, MO) supplemented with 0.25 mg/ml malachite green and 0.1% Tween-80 at pH 6.8. Cultures were grown until they reached the exponential growth phase (OD_600_ = 0.5), filtered through a 5 μm diameter pore membrane (Millipore, Inc., Billerica, MA) to remove cell clusters, and then centrifuged and resuspended in fresh media to obtain a single-cell suspension at OD_600_ = 0.01. PI (Sigma-Aldrich, St. Louis, MO) was added to the culture media and applied prior to seeding the cultures into the microdevice or while performing drug susceptibility testing to stain the DNA of dead cells. The final concentration of PI was 3 μM in this work.

The single-cell suspension was introduced into the prepared microdevice using a syringe pump at a flow rate of 50 μl/h. Flow was stopped upon visual confirmation of *M. smegmatis* entering into the culture chamber region of the microfluidic device using a 100× objective lens mounted on an inverted Zeiss Observer Z1 microscope. Then, a slower flow of 20 μl/h was applied for 30 min to rinse out excess bacterial cells that were not trapped. The microdevice was then placed into a temperature-controlled microscope incubation stage for continuous perfusion culture at 5 μl/h and long-term time-lapse microscopy imaging of cell growth. This cell seeding scheme is illustrated in Fig. S2.

### Microscopy and image analysis of *M. smegmatis* cultured in the microdevice

To image bacterial cells in the cell culture region, a Zeiss Observer Z1 microscope (Carl Zeiss AG, Oberkochen, Germany) equipped with an Orca Flash2.8 CMOS camera (Hamamatsu Photonics K.K., Hamamatsu-shi, Japan) and an incubation chamber with temperature and CO_2_ control was utilized. The cells were imaged with a 40× long-working-distance objective lens first and then with a 100× oil immersion objective lens to generate single-cell resolution images that were then used to analyze phenotypic changes in the cells. A custom image analysis algorithm using MATLAB^TM^ was developed to automatically analyze the bright-field and fluorescence images of *M. smegmatis* cells growing inside the microdevice to count the number of cells, as well as assess their morphology and length (i.e., phenotype). Measuring the length of the cells is important, as the length is known to reflect the bacterial metabolic status and response to drugs (e.g., drug resistance)^14^. As such, the fluorescence images were initially treated with threshold-based segmentation to locate initial regions of interest (ROIs). Then, using the bright-field images, the initial cell seeding regions were identified by threshold-based segmentation utilizing the previously defined ROI. Using the level set active contour algorithm, the contours of the seed regions were readjusted in the fluorescence images to obtain the final segmentation result. The longitudinal axis and directions were thereby determined, and the centerline of the longitudinal axis was used to measure the length of the *M. smegmatis* cells.

## Supplementary information

Supplementary Material
