# Supplementary material for: Development of single-cell-level microfluidic technology for long-term growth visualization of living cultures of Mycobacterium smegmatis
Source: Microsyst Nanoeng. 2021 May 20;7:37. doi: 10.1038/s41378-021-00262-1 (PMC8433163; doi:10.1038/s41378-021-00262-1)
Supplement: Supplementary file 1 — Supplementary Material [file 41378_2021_262_MOESM1_ESM.docx]

**Supplementary Information**

**Development of single-cell level microfluidic technology for long-term growth visualization of living cultures of *Mycobacterium smegmatis***

Han Wang‡^1^ · Gloria M. Conover‡^2,5^ · Song-I Han^3^ · James C. Sacchettini^2^ · Arum Han*^3,4^

^1^Department of Biomedical Engineering, School of Medicine, Tsinghua University, Beijing, 100084, China

^2^Department of Biochemistry and Biophysics, Texas A&M University, College Station, TX, 77843, USA

^3^Department of Electrical and Computer Engineering, Texas A&M University, College Station, TX, 77843, USA, Fax: 1-979-845-6259; Tel: 1-979-845-9686; E-mail: arum.han@ece.tamu.edu

^4^Department of Biomedical Engineering, Texas A&M University, College Station, TX, 77843, USA

^5^Current address: Department of Medical Education, Health Sciences Center, Texas A&M University, Bryan, TX 77807, USA

‡ H.W. and G.M.C contributed equally to this study.

**Figure S1**. Bulk culture growth dynamics of *M. smegmatis* cells grown in 7H9-ADC liquid media in shaking flasks over a 7-day period monitored by taking optical density OD_600_ measurements. Measurements were taken from diluted cultures to ensure accurate optical density readings. The ensemble data shows that the bacterial cultures reached saturation phase by 27 h after inoculation from a -80°C glycerol stock. Eight parallel cultures grown in individual flasks were used for this analysis.

**Figure S2.** Illustration of the cell preparation and seeding scheme into the microdevice (cyan). **(A)** *M. smegmatis* cells (green) were cultured overnight at 37^o^C in 10 ml of 7H9-ADC culture media in a plastic shaking flask. When the culture reached the exponential growth phase, it was filtered with 0.22µm polyvinylidene difluoride (PVDF) sterile 25 mm syringe filter to remove large cell clusters. The single-cell suspensions were then seeded into the microdevice using a syringe pump. **(B)** *M. smegmatis* cells follow the flow stream when introduced into the microdevice. **(C)** Single *M. smegmatis* cell (green) trapped in the shallow cell culture chamber regions (yellow).
